# Supplementary material for: Repeat expansion in a fragile X model is independent of double strand break repair mediated by Pol θ, RAD52, RAD54 or RAD54B
Source: Sci Rep. 2025 Feb 11;15:5033. doi: 10.1038/s41598-025-87541-3 (PMC11814403; doi:10.1038/s41598-025-87541-3)
Supplement: Supplementary file 1 — Supplementary Material 1 [file 41598_2025_87541_MOESM1_ESM.pdf]

Repeat expansion in a Fragile X model is independent of double strand break repair mediated by Pol θ, Rad52, Rad54l or Rad54b

Bruce E Hayward[1#], Geum-Yi Kim[1#], Carson J Miller[1#], Cai McCann[1], Megan G. Lowery[2], Richard D. Wood[2] and Karen Usdin[1,#]

[1] Section on Gene Structure and Disease
Laboratory of Cell and Molecular Biology
National Institute of Diabetes and Digestive and Kidney Diseases
National Institutes of Health, Bethesda, MD 20892
[2] The University of Texas MD Anderson Cancer Center
Department of Epigenetics & Molecular Carcinogenesis
PO Box 301429, Unit 1951, Houston, Texas 77230
[#] these authors contributed equally to this work.

[*] Corresponding author: **K. Usdin**
Tel: 301-496-2189
E-mail: karenu@nih.gov
Address: Building 8, Room 2A19
National Institutes of Health
8 Center Drive MSC 0830
Bethesda, MD 20892-0830

Supplemental Information

Supplemental Figures

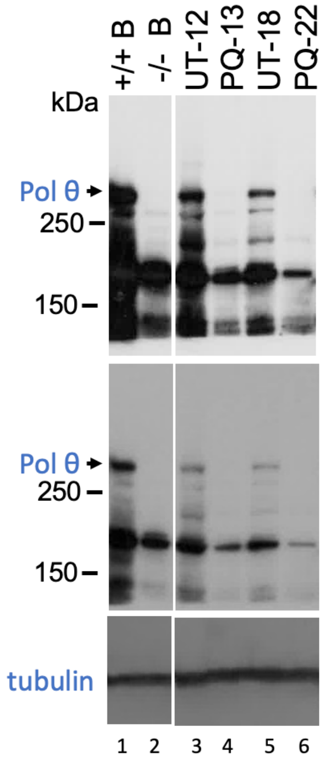

Supplemental Figure 1. Additional images relevant to Fig. 1b. An uncropped immunoblot of extracts from the indicated cell lines, using an antibody recognizing mouse Polθ. Extracts were prepared from B cells from *Polq*$^{+/+}$ mice (lane 1); B cells from *Polq*$^{-/-}$ mice (lane 2) were purified as described below. Extracts were prepared from *Polq*$^{+/+}$ mouse cell line WT-12 (lane 3); *Polq*$^{-/-}$ mouse cell line PQ-13 (lane 4); *Polq*$^{+/+}$ mouse cell line WT-18 (lane 5); *Polq*$^{+/+}$ mouse cell line PQ-22 (lane 6) as described in the main text. The top two panels show darker and lighter exposures of the same immunoblot. Intervening lanes containing irrelevant samples were excised from the immunoblot as indicated. The 50 kDa region of the gel was cut away and immunoblotted separately with an antibody against alpha tubulin, shown in the bottom panel.

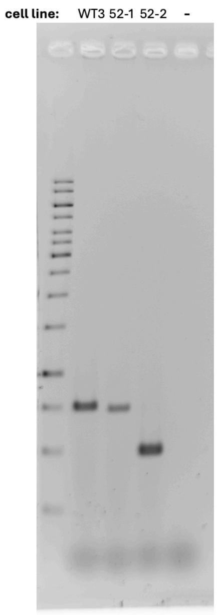

Supplemental Figure 2. Uncropped image from Fig. 2b showing PCR analysis of *Rad52* edited cell lines.

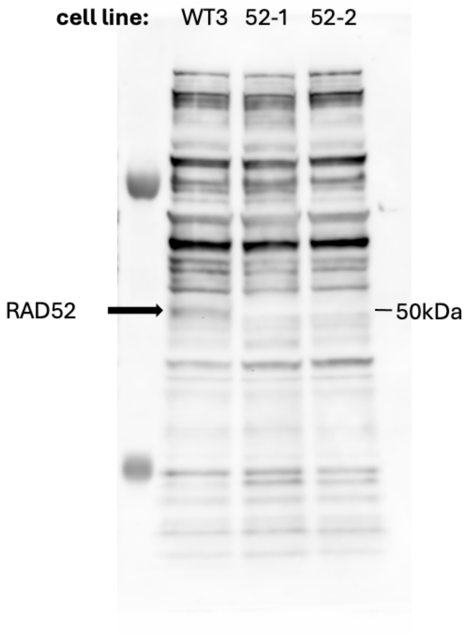

Supplemental Figure 3. Uncropped image corresponding to blot shown in Fig. 2c showing absence of RAD52 protein in the 52-1 and 52-1 lines.

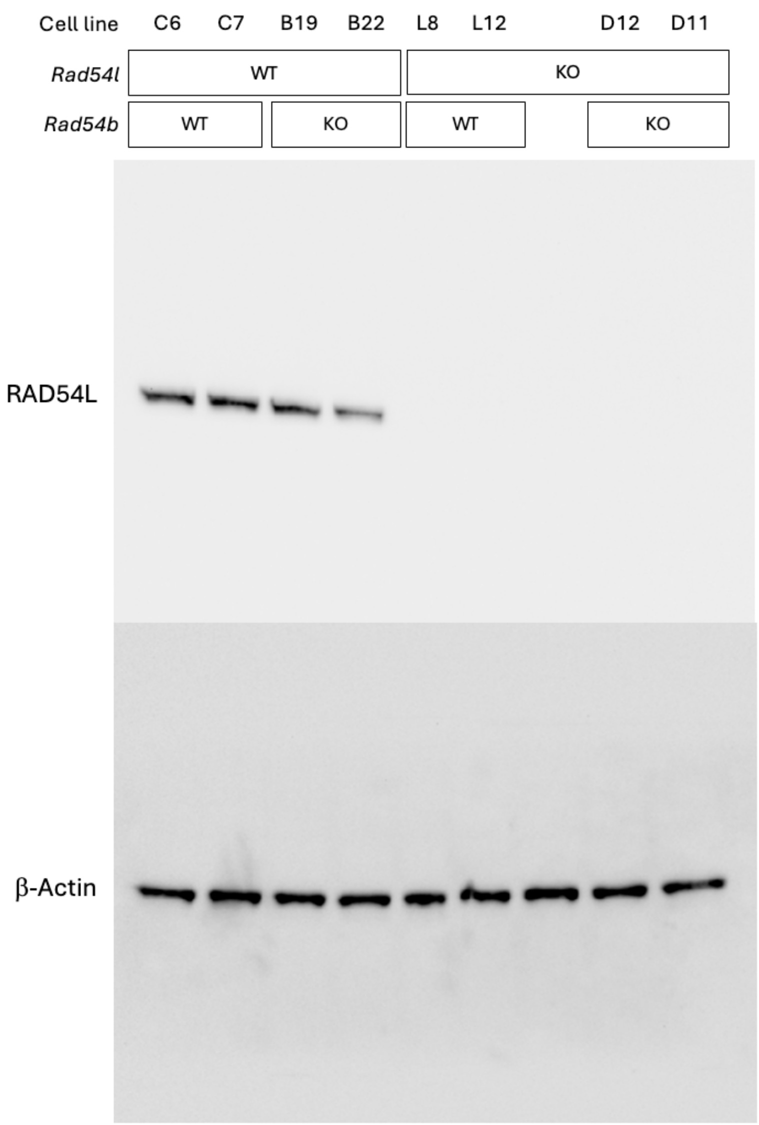

Supplemental Figure 4. Uncropped image corresponding to blot shown in Fig. 3c showing the presence of RAD54L in the C6, C7, B19 and B22 lines and its absence in the L8, L12, D12 and D11 lines. The unlabeled lane between L12 and D12 corresponds to a cell line that was not used for further analysis.

Supplemental Methods

Verification of Pol θ cell lines. Extracts were prepared by lysis of pellets from 5 x 10^6 cells. Cells were resuspended in buffer containing SDS, boiled for 10 min, sonicated, and insoluble material was removed by centrifugation. Samples (15 µL for B cells, 10 µL for other samples) were loaded on a 3-8% polyacrylamide gradient Criterion™ XT Tris-acetate protein gel and run with XT running buffer at 75 V for 180 min. Markers were All Blue Precision Plus Protein standards (Bio-Rad). Following transfer and drying of the membrane, immunoblotting used mouse monoclonal antibody against Pol θ. This antibody (153-5-1) was raised against a fragment of Pol θ QL [1]. Purified antibody (2.4 mg/mL) was used at 1:500 dilution in blocking buffer. Following washing, secondary goat anti-mouse HRP antibody was used at 1:10,000 dilution in 5% non-fat dried milk (CST) in TBS-T solution. The film was developed with Clarity plus reagent and exposed to x-ray film. A separate identical gel was loaded and stained with Revert total protein stain and imaged on an Odyssey Imaging System (LI-COR Biosciences) to confirm equal staining.

Preparation of mouse B-cell extracts. *Polq*$^{-/-}$ mice, originally derived by Shima *et al.* [2], were obtained from Jackson Laboratories as described and maintained on a C57BL/6J background [3]. Naïve mouse B cells were isolated from spleens, negatively sorted with anti-CD43 beads, and cultured with lipopolysaccharide and interleukin-4 as described [4]. Extracts were prepared after 72 h culture.

Supplemental References

1 Hogg, M., Seki, M., Wood, R. D., Doublie, S. & Wallace, S. S. Lesion bypass activity of DNA polymerase theta (POLQ) is an intrinsic property of the pol domain and depends on unique sequence inserts. *J Mol Biol* **405**, 642-652 (2011). https://doi.org:10.1016/j.jmb.2010.10.041

2 Shima, N., Munroe, R. J. & Schimenti, J. C. The mouse genomic instability mutation chaos1 is an allele of *Polq* that exhibits genetic interaction with *Atm*. *Mol Cell Biol* **24**, 10381-10389 (2004).

3 Yousefzadeh, M. J. *et al.* Mechanism of suppression of chromosomal instability by DNA polymerase POLQ. *PLoS Genetics* **10**, e1004654 (2014). https://doi.org:10.1371/journal.pgen.1004654

4 Minkah, N. *et al.* Absence of the uracil DNA glycosylase of murine gammaherpesvirus 68 impairs replication and delays the establishment of latency in vivo. *J Virol* **89**, 3366-3379 (2015). https://doi.org:10.1128/JVI.03111-14
